# Are We Sentenced to Pharmacotherapy? Promising Role of Lycopene and Vitamin A in Benign Urologic Conditions

**DOI:** 10.3390/nu14040859

**Published:** 2022-02-18

**Authors:** Piotr Kutwin, Piotr Falkowski, Roman Łowicki, Magdalena Borowiecka-Kutwin, Tomasz Konecki

**Affiliations:** 1 1st Department of Urology, Medical University of Lodz, 90-549 Lodz, Poland; pfalkowski1@gmail.com (P.F.); roman.lowicki@umed.lodz.pl (R.Ł.); tomasz.konecki@umed.lodz.pl (T.K.); 2 Department of Invasive Cardiology and Cardiac Arrhythmias, Medical University of Lodz, 90-549 Lodz, Poland; magdabor88@gmail.com

**Keywords:** carotenoids, lycopene, urology, benign prostate hyperplasia, urinary tract infections, urolithiasis, chronic prostatitis

## Abstract

Benign prostatic hyperplasia, urolithiasis, recurrent urinary tract infections, and chronic prostatitis are diseases that are commonly diagnosed worldwide. Carotenoids, including lycopene, are widely available in fruits and vegetables, and it is postulated that they can be used in the prevention and treatment of benign urological conditions. The aim of this review is to familiarize doctors and their patients with the current knowledge on carotenoids and their conversion products in selected urological diseases. Most of the experimental and clinical trials show a moderate effect of lycopene and vitamin A on studied parameters. Lycopene was shown to improve the IPSS score in BPH patients, and alleviate symptoms in those with chronic prostatitis. Intake of Vitamin A was associated with decrease of urinary tract reinfection rates. In studied rat models retinol also decreased urolithiasis formation. Although the results of the cited studies are generally promising, it is evident that more detailed and extensive research must be done in this field of medicine.

## 1. Introduction

Both the scientific community and patients have become increasingly interested in natural medicine in the last few years. Carotenoids, seleno-compounds, and polyphenols are phytochemicals that have been assessed in many benign and malignant medical conditions. Only six (α- and β-carotene, β-cryptoxanthin, lutein, lycopene, and zeaxanthin) out of the seven hundred known carotenoids are detectable in considerable amounts in the human diet [1]. Insight into the absorption, distribution, and processing of lycopene may lead to better interpretation of earlier epidemiologic findings and investigations. The uptake of carotenoids in the human organism takes place in the proximal part of the small intestine. In their micellar forms, consumed carotenoids, including lycopene, are absorbed actively into the intestinal mucosa cells [2]. The absorption of vitamin A and carotenoids is controlled by β-carotene 15,15’ dioxygenase-1 (BCO1) in a negative feedback manner. BCO1 converts beta-carotene to retinol, which in turn leads to higher expression levels of homeobox transcription factor (ISX). ISX connects to DNA binding motifs upstream of the SCARB1 genes, which trigger cell production of membrane proteins that facilitate intestinal absorption of vitamin A and carotenoids [3]. Their absorption is largely influenced by the amount of lipids in the meal. Lycopene distribution is not equal among tissues. Adipose tissue and liver are its two main body reservoirs. The highest concentration levels of lycopene are noted in human testes and adrenal glands [4].

**Figure 1:**
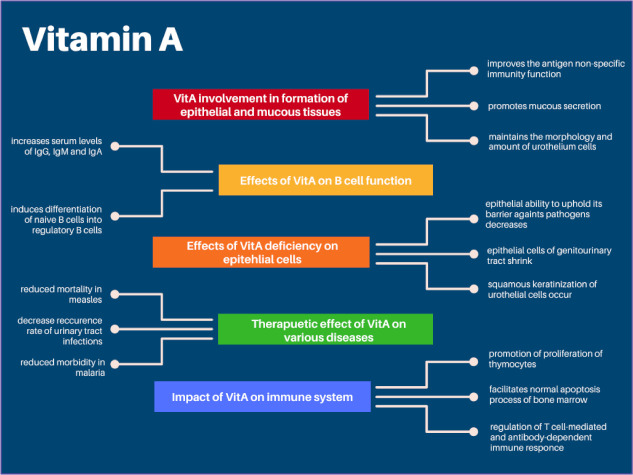
Vitamin A’s regulatory role in immune response and its therapeutic effect. Based on Huang et al. [6].

Carotenoids such as β-carotene can be transformed into vitamin A (VitA) and demonstrate its biological activity within the organism. VitA already has a well-established position in embryogenesis, reproduction, and ophthalmology [5]. However, not all of its mechanisms of action are fully understood, and new areas of medicine, which may be influenced by retinol, are still under investigation. Proposed mechanisms of action of VitA and its regulatory role in different immunological pathways were studied widely by Huang et al. (Figure 1).

**Figure 2:**
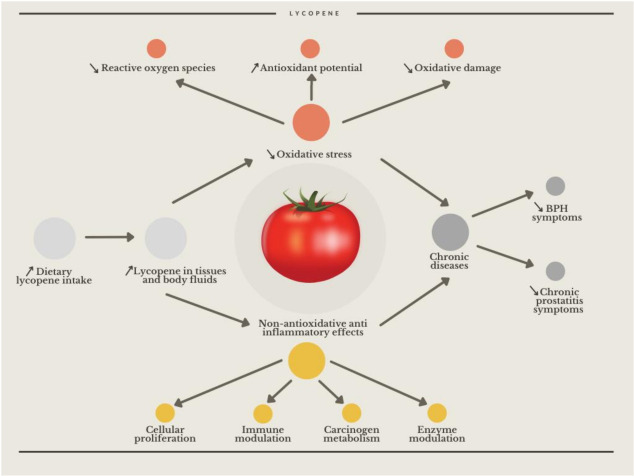
Proposed anti-inflammatory mechanisms for lycopene in preventing benign urological diseases. Based on figures from Agarwal et al. [9].

Dulińska-Litewska et al. focused on molecular aspects of carotenoid action in their extensive review, studying the enzymes responsible for carotenoid metabolism pathways. In humans, there are two enzymes—BCO1 and β-carotene 9’,10’ dioxygenase-2 (BCO2)—involved in converting carotenoids into bioactive compounds [7]. Genetically modified mice lacking BCO1, which are unable to synthesize retinol, present with multipoint disruptions in prostatic androgen receptor signaling and many other disorders, e.g., changes in steroid metabolism [8]. The proposed hypotheses that explain its mechanisms of action can be divided into two groups: nonoxidative and oxidative (Figure 2).

**Figure 3:**
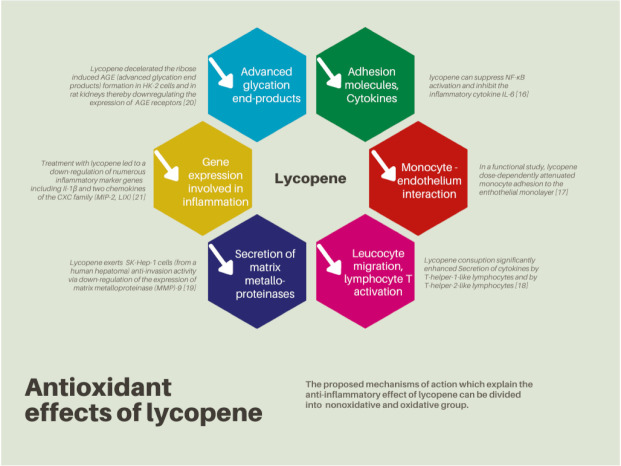
Antioxidant effects of lycopene. Based on figures from Mozos et al. [22].

The regulation of gap-junction communication in fibroblast cells [10], prevention of beta-carotene 9’,10’-oxygenase suppression [11], regulation of the cell cycle [12], modulation of the liver metabolizing enzyme cytochrome P4502E1 [13], reduction of cellular proliferation induced by insulin-like growth factors [14], enhancement of the total percentage of T-cells, and increased NK-cell activity [15] are proposed nonoxidative mechanisms responsible for the anticarcinogenic effects of lycopene (Figure 3) [16,17,18,19,20,21].

Lycopene has demonstrated greater antioxidant features compared to other carotenoids, such as β-carotene, lutein, and zeaxanthin [23]. Lycopene in fruits and vegetables is usually present as all-trans isomers [24]. However, lycopene is unstable in air and water [25]. Currently, approaches for lycopene preparation include extraction from tomatoes, chemical synthesis, and microbial fermentation [26,27].

According to the updated definition, oxidative stress is “an imbalance between oxidants and antioxidants in favor of the oxidants, leading to a disruption of redox signaling and control and/or molecular damage” [28]. Reactive oxygen species are the natural products of ongoing body processes involving oxidative metabolism, mitochondrial bioenergetics, and immune function [29]. Oxidative stress has been linked with many chronic diseases, including cancer, and with acceleration of aging processes [30]. Vegetables and fruits contain many active compounds showing anti-inflammatory and antioxidant abilities, so their intake may have protective effects on many benign urological conditions by maintaining and restoring oxidative homeostasis. Removing free radicals demonstrates a protective effect on cells from oxidation caused by their own components [31].

**Figure 4:**
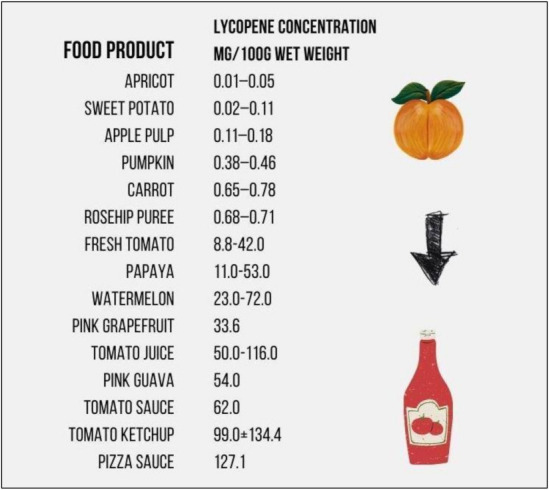
Lycopene content of fruit, vegetables, and processed tomato products [32,33].

Lycopene gives the red color to plants, and it is found in several fruits and vegetables, including tomatoes, strawberries, pink grapefruits, and watermelons [32,33]. The concentration levels of lycopene in selected food products are presented in Figure 4.

There is still no data regarding the ideal daily intake of lycopene. However, a daily intake of 6 mg is sufficient to obtain antioxidant properties [34]. It is noteworthy that food processing improves the absorption bioavailability of lycopene in tomato-based foods compared to raw tomato juice [35].

Benign urological conditions such as benign prostatic hyperplasia (BPH), urolithiasis, recurrent urinary tract infections (rUTI), and chronic prostatitis are diseases that are commonly diagnosed worldwide. In 2010, BPH affected over 210 million men [36]. This made it a major health problem in older, as up to 50% of men over the age of 50 experience lower urinary tract symptoms (LUTS) due to enlarged prostate [37]. Rates of nephrolithiasis ranged from 7–13% in North America, 5–9% in Europe, and 1–5% in Asia [38]. In the United States, patients suffering from prostatitis alone are estimated to contribute 2–8 million outpatient visits per year [39], while those with urinary tract infections, in 2007 alone, needed 10.5 million ambulatory consultations [40]. All these numbers show the huge burden of urological diseases, which are likely to grow in number even more as societies age. In recent years, many patients have turned to alternative medicines to improve their general quality of life and prevent diseases [41]. The aim of this review is to familiarize doctors and their patients with current knowledge on carotenoids in selected benign urological conditions and help them to start adding phytochemicals to treat and prevent diseases.

## 2. Benign Prostatic Hyperplasia

Benign prostatic hyperplasia (BPH) is a proliferative disorder of the prostate gland arising from its epithelial cells and smooth muscle within the transitional zone [42]. Increased smooth muscle tone and obstruction of the urethra lead to LUTS [43]. Prostate growth is induced by androgen stimulation and dihydrotestosterone (DHT), produced from testosterone by 5-alpha-reductase, which is a hormone that primes this phenomenon [44].

After lifestyle modifications, which are a first-line treatment in mildly symptomatic disease, administration of medications is the mainstay in the treatment of most men with symptomatic BPH. For this purpose, two drug classes, i.e., 5-alpha-reductase inhibitors and Alpha-blockers have been adopted as the standard of care [45]. As these drugs are not free of adverse events, including a loss of libido, erectile dysfunction [46], and dizziness [47], it is advisable to seek alternative methods to treat and prevent BPH.

Lycopene appears to reach high levels in the prostate and human semen [48,49]. However, the mechanism itself by which lycopene is accumulated in prostatic tissue and excreted into semen remains unknown. High concentration levels of lycopene in prostatic tissue are linked with the prevention of pathologies, such as BPH. These actions are thought to be mediated through various mechanisms, including the inhibition of 5-alpha-reductase expression [50].

### 2.1. BPH Epidemiological Studies

The largest observational study, which primarily focused on dietary patterns and BPH occurrence, was performed by Tavani et al., who included 2820 men. Of this number, 1369 suffered from BPH. The authors concluded that the risk of BPH significantly decreased with an increasing intake of carotene, vitamin C, and iron. The intake of lycopene or zeaxanthin did not impact BPH incidence. These results contradict those of interventional studies conducted so far. However, as the authors noted, no uniform case definition of the disease has been established and only surgically treated men with BPH were included in this study, which is a potential reason for the lack of dependency between lycopene consumption and BPH prevalence [51].

Kristal et al. examined dietary risk factors for incidence of benign prostatic hyperplasia in 4770 Prostate Cancer Prevention Trial placebo-arm participants who were free of BPH at baseline. BPH was assessed in this group of men over a 7-year period. The authors found that a dietary pattern low in vegetables and protein, and high in fat and red meat, was associated with the development of symptomatic BPH. There was also a weak association between lycopene, zinc, and supplemental vitamin D intake and decreased BPH occurrence [52].

### 2.2. BPH Experimental Studies

A few in vitro studies have shown that lycopene inhibits the proliferation of benign prostate epithelial cells [53] and suppresses inflammatory cascade [54]. The mechanism responsible for this effect might be the inhibition of 5-alpha-reductase and basal inflammatory signaling, assessed in benign prostate tissue of rats. However, a study performed by Herzog et al. did not show the influence of lycopene administration on prostate growth in young rats [21].

An experimental model showed that a combination of Selenium (Se), Serenoa Repens (SeR), and Lycopene (Ly) effectively reduces oxidative stress, prostate inflammatory response, and histological features [54]. Another study performed by Minutoli et al. also investigated the influence of SeR, Se, and Ly on the microscopic effects of supplementation on BPH tissue. Administering SeR, Se, and Ly significantly blunted prostate growth. Moreover, the combination of SeR–Se–Ly was most effective in reducing prostate enlargement and growth by 43.3% in treated animals [55].

Both in vitro and clinical studies indicate that lycopene potentially inhibits BPH progression. Kim et al. administered 30 mg of lycopene per day for three weeks before radical prostatectomy to 32 patients diagnosed with prostate cancer (PCa). Later on, they investigated the impact of lycopene consumption on histopathological changes found in prostate specimens assessed post-surgically. They revealed that lycopene induced apoptosis in cancer-free BPH tissue. Apoptosis affected both epithelial and myoepithelial cells [56].

Lycopene was not the only carotenoid that demonstrates antioxidant properties tested in pre-clinical trials aimed at BPH treatment. Hou et al. investigated astaxanthin (AST) in the BPH rat model. They studied the effects of tested carotenoid on prostate weights, superoxide dismutase (SOD) activity, and testosterone and dihydrotestosterone levels depending on the dose of the administered substance (20 mg/kg, 40 mg/kg and 80 mg/kg). The most pronounced decline in prostate weights was observed after delivering 80 mg/kg of AST, while noticeable changes in hormone levels and SOD activity started from the administered dosage of 40 mg/kg; they concluded that AST has an inhibitory effect on testosterone-induced rats [57].

### 2.3. BPH Clinical Studies

The inhibitory effect of lycopene observed in in vitro studies has been further assessed in clinical settings. Coulson et al. assessed the effectiveness of 3-month treatment of men with lycopene preparations. A significant reduction in the IPSS (International Prostate Symptom Score) and day- and night-time urinary frequency compared to the placebo group was observed, confirming the usefulness of the preparations in men with histologically diagnosed BPH [58]. Similar conclusions were drawn by Li et al., who assessed the IPSS, quality of life (QoL) score, prostate volume, prostate specific antigen (PSA) level, maximum urinary flow rate, and postvoid residual volume (PVR). Except for the prostate volume, all the other evaluated parameters improved after four months of lycopene intake [59].

Swartz et al. investigated the effects of lycopene supplementation on men diagnosed with BPH. Forty patients with histologically confirmed BPH were randomized to receive either lycopene at a dose of 15 mg/d or placebo for six months. The six-month lycopene supplementation decreased PSA levels, improved IPSS score symptoms, and stopped further enlargement of the prostate. The study showed no interference between lycopene and PSA levels, which is relevant to the issue of PCa detection during the long-term intake of supplements [60].

Large randomized, double-blinded studies confirming the efficacy of lycopene in BPH are still unavailable. Despite these objections, lycopene intake should be considered an effective treatment for mildly symptomatic BPH patients. The study on carotenoids carried out so far is one of the best examples of phytochemical effects on the disease (Table 1).

## 3. Prostatitis

Prostatitis is a common urologic condition that in 1999 was subdivided into four categories—acute bacterial prostatitis, chronic bacterial prostatitis, chronic non-bacterial prostatitis/chronic pelvic pain syndrome (CPPS), and asymptomatic inflammatory prostatitis [63]. CPPS accounts for 90–95% of all prostatitis cases. Patients usually report symptoms of discomfort in the pelvis, genital, and suprapubic area, urinary symptoms, and sexual dysfunction [64].

The etiopathogenesis of CPPS is still unclear. Since no invading infectious agent has been identified, many hypotheses have been put forward to explain the CPPS etiopathogenesis. They include defective urothelial integrity and function, autoimmune triggered inflammation state, endocrine imbalances, pelvic floor muscle spasm, peripheral and central sensitization, and psychosocial conditions [65]. In the latest research performed by Zhou et al., increased oxidative stress and oxidative damage induced by chronic bacterial prostatitis were found in patients, and such phenomena were closely related to the course of the disease [66].

CPPS is considered a challenge in outpatient clinics. As conventional treatment of CPPS seems hardly effective, more attention has been paid to alternative treatments, including carotenoid therapy.

### 3.1. Prostatitis Experimental Studies

The suppressive effect of antioxidant supplement (Prosta-Q) on inflammatory processes in prostatitis was assessed by Shahed et al. The product includes zinc, quercetin, cranberry, saw palmetto, bromelain, and papain. It is speculated that oxidative stress may be a key pathway in some men with CPPS, which can be targeted with antioxidant therapy [67]. Lycopene, which downregulates inflammatory regulators such as cytokines, enzymes, and transcription factors in cell culture systems [68], is also known to decrease expression markers for immune cell infiltration in rats’ prostate tissue [21].

The synergistic effect of chronic bladder pain (CBP) treatment with lycopene and fluoroquinolones was demonstrated by Han et al. Their analysis of microbiological cultures of the prostate and urine as well as histological findings showed that the addition of lycopene to antibiotic treatment is associated with a statistically significant decrease in bacterial growth and improved prostatic inflammation compared with the ciprofloxacin group [69].

Morgia et al. evaluated the efficacy of the SeR–Se–LY combination in reducing chronic inflammation in patients with benign prostatic hyperplasia and/or prostate intraepithelial neoplasia or atypical small acinar proliferation (PIN/ASAP). This was a multicenter study involving nine Italian urological centers between January 2009 and December 2010. The influence of the test substances on the inflammatory state was measured by histo-biochemical methods. The anti-inflammatory effects were indirectly measured by evaluating the density of T-cells (CD3, CD8), B-cells (CD20), and macrophages (CD68). At the six-month follow-up, there were statistically significant reductions of extension and grading of inflammatory infiltration, mean values of CD20, CD3, and CD68, and mean PSA value in a group of patients with chronic prostatic inflammation taking SeR–Se–LY compared with the control group. It was concluded that patients with bladder outlet obstruction could benefit from this therapy acting on the inflammatory component of BPH [70].

### 3.2. Prostatitis Clinical Studies

The effects of SeR–Se–LY on IIIa CPPS were compared to Serenoa repens alone in a randomized study performed by Morgia et al. After eight weeks of treatment, S. repens + selenium and lycopene were found to ameliorate symptoms associated with chronic prostatitis, providing significant improvement in voiding dysfunctions compared to S. repens alone. As the treatment was safe and well tolerated, the authors pointed out its usefulness when long-term therapy is required [71].

Cai et al. assessed the influence of adding Serenoa repens, selenium, lycopene, bromelain, and methyl-sulfonyl-methane extracts to standard levofloxacin therapy for chronic bacterial prostatitis. They found that combination therapy had a significant effect on all three evaluated scores (QoL, NIH-CPSI, and IPSS) compared to antibiotic treatment alone. Moreover, as the authors pointed out, no adverse drug reactions have led to high compliance with the experimental protocol [72].

Based on evidence from observational and experimental studies, lycopene shows therapeutic activity against chronic prostatitis. Although its effect was assessed as a support to standard antibiotic treatment, the results of the studies are encouraging. Taking into account chronicity and the recurrent nature of the disease, which demands long-term drug usage, carotenoid supplementation seems to be a reasonable choice for such patients (Table 2).

## 4. UTI

Urinary tract infection (UTI) is one of the most common bacterial infections affecting women [73]. Recurrent urinary tract infection (rUTI) is frequently defined as two or more episodes in the last six months or at least three episodes in the last 12 months [74]. The risk of developing a UTI in a lifetime has been estimated to be above 50% [75], with 25% having a recurrence [76]. Urinary tract infection may be confined to the lower urinary tract (cystitis), or it may also affect the upper urinary tract (acute pyelonephritis). A variety of oxidation products are found in urine participating in local and systemic oxidative stress reactions [77]. Furthermore, UTI severely increases oxidative stress in patients [78].

Prevention strategies, including the replacement of vaginal estrogens, taking D-mannose supplements, nitrofurantoin applied at a daily dose of 50 mg methenamine salts, and cranberry products, are major components of rUTI care. The management goal is to significantly reduce UTIs. However, it is not always possible to eliminate the infection completely despite strict adherence to medical recommendations [79].

There are a few described mechanisms by which the protective effect of vitamin A on the urinary tract can be observed. Vitamin A increases both immune response efficacy to infection once the epithelial barrier has been disrupted [80] and non-specific immunity, by supporting the physical and biological integrity of epithelial tissue as the first barrier to infection [81]. Furthermore, it can provide a more effective barrier against infection by restoring normally differentiated epithelium that coats the urinary tract, preventing pathogen adhesion [82].

### 4.1. UTI Experimental Studies

Munday et al. studied vitamin A deficiency in rat models. They found pyelonephritis in 68% and cystitis in 66% of rats after 34 weeks of the experiment. The vitamin A deficiency in rats led to squamous metaplasia that was confined to the transitional epithelium, suggesting susceptibility of this epithelial type to vitamin A deficiency. Squamous metaplasia was thought to be the likely reason for bacterial infections observed within the rats’ urinary tract [83].

### 4.2. UTI Clinical Studies

Kahbazi et al. studied the addition of vitamin A to antibiotic therapy in the acute phase of pyelonephritis (APN). The results showed that oral vitamin A treatment during APN resolves some of the clinical symptoms of UTI more quickly and reduces renal scarring following APN compared to antibiotics alone. This was the first report to present the antipyretic and anti-urinary frequency effects of vitamin A [84]. It is thought that this effect arises from retinol’s hormone-like activity as a growth factor for epithelial cells, and vitamin A’s re-epithelialization properties of damaged mucosal surfaces [85].

**Figure 5:**
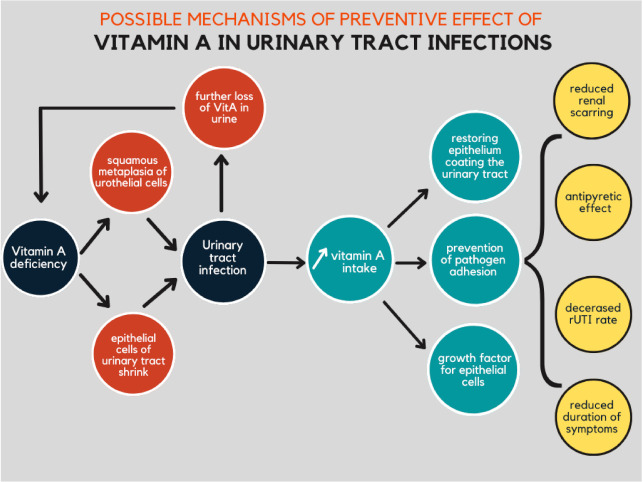
The possible cascade leading to urinary tract infection and preventive effects of vitamin A supplementation in the urinary tract infections.

Yilmaz et al. investigated the influence of vitamin A supplementation on recurrent urinary tract infection. UTI recurrence rates during the 12-month follow-up significantly decreased after a single dose of vitamin A given when the patients were enrolled in the study. This decrease was notably more prominent during the first six months [86]. The consequences of Vitamin A deficiency on urinary tract and possible effects of VitA supplementation are shown in Figure 5.

Although evidence of vitamin A’s effect on urinary tract infection is sparse, research thus far confirms its supportive role in managing acute and recurrent UTI (Table 3). To draw any final conclusions concerning carotenoid efficacy in the treatment and prevention of UTI, additional well-powered studies are still needed.

## 5. Urolithiasis

Urolithiasis can form anywhere in the urinary tract, and it is the cause of potentially acute or chronic states depending on the location and stone size. The mainstay of treatment is focused on the lithotripsy of existing calculi. Nutritional management is designed to both dissolve particular urinary stones (e.g., urate) and reduce the risk of recurrence. Urolithiasis prevention is based on decreasing the supersaturation of stone compounds in urine. Current paradigms are based on evidence linking urine composition and dietary habits [87].

Although a complete picture of the pathophysiological mechanisms involved in stone process formation is still unclear, experimental and clinical studies provide evidence for the production of reactive oxygen species and the development of oxidative stress in patients with stone disease [88].

### Urolithiasis Experimental Studies

Naghii et al. studied the effects of combining natural antioxidants such as vitamins A, C, E, and B6, and Zinc and Selenium on the development of nephrolithiasis in rat models. The effect of treatment was assessed by counting the number of crystal deposits in the microscopic fields in the kidney specimens. Urolithiasis was completely absent in the kidneys of the antioxidant group, showing the beneficial effects on the prevention and elimination of calculi in the rat urinary tract. It was presumed that the impact of these nutrients on the prevention and disruption of kidney stones may be, at least, in part due to an antioxidant effect [89].

The protective effect of vitamin A supplementation against calcium-oxalate stone formation is thought to be due to the increased production of glycosaminoglycans and glycoproteins, which both inhibit crystallization. An experimental study performed by Grase et al. found lowered concentration of urinary glycosaminoglycans and zinc in rats fed with a vitamin A-deficient diet [90].

The effect of vitamin A supplementation on calcium-oxalate stone formation in rats was studied by Bardaoui et al. They showed that vitamin A given at the rate of 20 times the standard ration improves the renal function by restoring the glomerular filtration rate. Moreover, an increase in the urinary pH and higher excretion of citric acid was observed. The results indicate the beneficial effects of vitamin A supplementation against renal stone formation [91].

In another experimental study performed on rats, Munday et al. studied the influence of a vitamin A-deficient diet on animals’ health status. Examination of rats that completed the 34-wk experiment revealed urolithiasis in 27%, and nephrolithiasis in 5% [83]. Although it was noted that the exact mechanism of vitamin A deficiency leading to stone formation remains unclear, keratin debris-promoting calculus formation [92] and alterations in urine composition [90] might be the possible patho-mechanism.

Supplementing carotenoids could be considered an effective option to protect against stone deposition in the urinary tract, especially when no apparent reason for stone formation is noticeable. As Salky et al. showed, when idiopathic renal stone genesis is suspected, vitamin A deficiency could be a possible reason for this state [93].

Evidence assessing the effects of carotenoids on urolithiasis is mainly based on experimental rat models, and its direct transposition into the human organism might be an oversimplification of this clinical problem. Nevertheless, current studies favor vitamin A use, showing its multipoint preventive mechanism against stone formation (Table 4).

## 6. Conclusions

Recent lines of evidence from pre-clinical and clinical studies indicate that lycopene, tested as a solitary drug or in combination with other substances, has the potential to play a beneficial role in the alleviation of BPH and chronic prostatitis symptoms. Vitamin A, which is formed in the course of metabolic processes from carotenoids, decreases chances of reinfection rate and stone formation in the urinary tract.

Although the results are generally promising, it is evident that more detailed and extensive research has to be done on the efficacy of carotenoids and their conversion products in the treatment and prevention of all benign urological conditions, with special insight into urinary tract infection and urolithiasis, as these two groups of diseases have been of interest in only a few studies so far. The lack of standardized protocols and outcome measurements, and the relatively small study sizes may be the most relevant causes of some outcome bias. High-quality randomized controlled trials (RCTs) that investigate the effectiveness of lycopene and vitamin A in the prevention and treatment of benign urological conditions are needed. Although many studies have proven its potential usefulness, currently there is a lack of sufficient evidence to support, or refute, the use of lycopene and retinol in any of the diseases mentioned in the manuscript.

In order to observe the action effects of lycopene and vitamin A, large-scale clinical trials must be carried out to obtain statistically significant results. Patients should be randomized to receive different daily dosages of the tested substance over a specific period, and the outcomes measured should be specific for each condition assessed. For this purpose, investigators should consider specific populations, accurate measurement of consumption, a clear definition of urologic diseases, and other confounding factors.

Overall, these results should encourage the development of promising individualized nutritional strategies to tackle many chronic urological non-oncological diseases, as pharmacotherapy or surgery should not remain the only available therapeutic options for patients.

## Figures and Tables

**Table 1:** Characteristics of studies assessing lycopene effects on benign prostate hyperplasia treatment and prevention.

Study	Year	Studied Population	Intervention	Results	Ref.
Carrasco et al.	2021	20 men aged > 50 allocated into one of two groups: healthy-men (*n* = 10) and BPH (*n* = 10)	For a period of 30 days, both groups ingested 20 mL of lycopene daily	PSA levels and reported symptoms improved in the BPH group; however, the difference was not statistically significant. The overall antioxidant status was significantly increased in the healthy-men group (*p* < 0.05)	[61]
Coulson et al.	2012	57 men aged 40–80 years with BPH, randomized into intervention and placebo groups	For three months, the patients in the intervention group received one capsule per day of herbal preparation (*n* = 32). The patients in the second group were given a placebo (*n* = 25)	The IPSS score in the intervention group decreased by 36%, while in the placebo group, the reduction of symptoms reached 8% (*p* < 0.05)	[58]
Li et al.	2019	120 patients diagnosed with BPH	All the participants consumed lycopene tablets 500 mg twice a day for 1 year	After two and four months of treatment, there was a significant improvement in the IPSS, QoL score, and Qmax compared with the baseline. The prostate volume did not change before and after medication intake	[59]
Morgia et al.	2014	225 patients aged 55–80 years	Participants were assigned to group A, consuming SeR–Se–Ly, group B, taking tamsulosin, or group C, receiving both products	The decrease in IPSS score was significantly more noticeable in Group C than group A (*p* < 0.05) and group B (*p* < 0.01). The changes in IPSS score and Qmax levels were more pronounced for combination therapy versus monotherapies (<0.05).	[62]
Schwarz et al.	2008	40 patients with histologically proven BPH without coexisting prostate cancer	Patients were assigned to lycopene receiving group at a dose of 15 mg/d for six months, or a placebo group	After six months of lycopene intake, PSA levels significantly decreased (*p* < 0.05). Meanwhile, there was no change in the placebo group. The levels of lycopene assessed in plasma increased after lycopene intervention (*p* < 0.0001), whereas the concentration of other carotenoids did not change.	[60]

BPH—benign prostatic hyperplasia; PSA—prostatic specific antigen; IPSS—International Prostate Symptom Score; LUTS—lower urinary tract symptoms; QoL—quality of life; Qmax—maximum flow rate; SeR–Se–Ly—Serenoa Repens–selenium–lycopene; PVR—postvoid residual volume.

**Table 2:** Characteristics of studies on chronic prostatitis treatment in which lycopene was used as a solitary drug or in combination with other substances.

Study	Year	Studied Population	Intervention	Results	Ref.
Cai et al.	2016	79 patients suffering from CBP	The participants were assigned to one of two groups: Group A taking levofloxacin 500 mg once daily for two weeks with lycopene and methylsulfonylmethane addition; Group B receiving only the antibiotic	In group A there was a significant improvement in NIH-CPSI (−17.6 ± 2.65) and IPSS (−12.2 ± 2.33) scores versus Group B (mean difference: −9 ± 1.82; −8.33 ± 1.71, respectively)	[72]
Morgia et al.	2010	102 patients suffering from IIIa CP/CPPS, aged 23–49 years	Patients were randomly assigned into two groups: group A receiving Profluss (*S. repens*, selenium, and lycopene) or group B taking *S. repens* alone for two months	The NIH-CPSI score significantly improved (*p* < 0.001) in both groups; the decrease in IPSS score and improvement in the maximum peak flow rate was seen in both arms, but was more pronounced in group A. The decrease of PSA and WBC count (*p* < 0.007) was only reported in group A	[71]
Morgia et al.	2013	168 patients suffering from BPH submitted to prostate biopsy for PCa suspicion. Two additional cores were taken for PCI evaluation	The first group consisted of 108 participants with histological diagnosis of PCI randomized to Profluss group (I) or to control group (Ic). The second group consisted of 60 participants with histological diagnosis of BPH, randomized to Profluss + α-blocker treatment group (II) or to the control group (IIc)	Alleviation of inflammatory state, decrease in mean values of interleukins (CD20, CD3, CD68), and mean PSA levels in group I compared to group Ic. The extension and grading of inflammatory state in group II were also decreased compared to IIc, but not statistically significantly. A statistically significant difference in interleukin levels (CD20, CD3, CD68, CD8) was reported in group II compared to IIc	[70]

CBP—chronic bladder pain; NIH-CPSI—National Institutes of Health—Chronic Prostatitis Symptom Index; IPSS—International Prostate Symptom Score; CP—chronic prostatitis; PCI—prostate chronic inflammation; CPPS—chronic pelvic pain syndrome; WBC—white blood cells; PCa—prostate cancer.

**Table 3:** Characteristics of studies assessing vitamin A effects on urinary tract infection treatment and prevention.

Study	Year	Material	Intervention	Results	Ref.
Kahbazi et al.	2017	90 females aged 2–12 years diagnosed with UTIs and the first episode of APN	Participants were randomized into two groups: in addition to antibiotics the intervention group was given 10 days of oral vitamin A while the control group received 10 days of placebo	Duration of symptoms (fever, urinary frequency, and poor feeding) was significantly reduced in the intervention group. The second 99mTc-DMSA scan revealed worsening of patients’ kidney status in 22.2% of participants in the vitamin A group and 44.7% of patients in the placebo group (*p* = 0.003)	[84]
Yilmaz et al.	2007	24 patients with uncomplicated rUTI were included	Patients were randomized into two groups: the first receiving a single dose of 200,000 IU vitamin A in addition to antibiotic treatment and the second being a control group	In the six months after treatment, the chance of suffering rUTI reduced from 3.58 to 0.75 in the intervention group. UTIs were statistically less frequent during the six months follow-up after vitamin A supplementation compared to the control group	[86]

UTI—urinary tract infection; rUTI—recurrent urinary tract infection; APN—acute pyelonephritis.

**Table 4:** Characteristics of studies assessing vitamin A effects on urolithiasis prevention.

Study	Year	Material	Intervention	Results	Ref.
Bardaoui et al.	2009	24 male Wistar rats were randomized into three groups	Group A was fed a normal diet. Group B was given a lithogenic diet. Group C received a lithogenic diet for three weeks then a vitamin A supplemented diet for the three last weeks.	The glomerular filtration rate and the urinary excretion of citric acid—which fell in group B—were restored in group C.	[91]
Munday et al.	2009	100 female Sprague–Dawley rats	Rats were randomized to a group fed with diets that included a vitamin premix or a group fed with albumin or milk powder	Examination of the 44 rats fed the albumin diet (vitamin A deficient diet) that completed the 34-wk experiment revealed urolithiasis in 27%, and nephrolithiasis in 5%	[83]

## Data Availability

Not applicable.
